# GPR120 Inhibits RANKL-Induced Osteoclast Formation and Resorption by Attenuating Reactive Oxygen Species Production in RAW264.7 Murine Macrophages

**DOI:** 10.3390/ijms221910544

**Published:** 2021-09-29

**Authors:** Cynthia Sithole, Carla Pieterse, Kayla Howard, Abe Kasonga

**Affiliations:** 1 Department of Physiology, Faculty of Health Sciences, University of Pretoria, Pretoria 0001, South Africa; u15039499@tuks.co.za (C.S.); carla.pieterse@yahoo.com (C.P.); 2 Division of Clinical Pharmacology, Faculty of Medicine and Health Sciences, Stellenbosch University, Cape Town 8000, South Africa; kaylahoward101@gmail.com

**Keywords:** osteoclasts, GPR120, reactive oxygen species, resorption

## Abstract

Osteoclasts are large, multinucleated cells that are responsible for the resorption of bone. Bone degenerative diseases, such as osteoporosis, are characterized by overactive osteoclasts. Receptor activator of nuclear factor-κB (NF-κB) ligand (RANKL) binding to its receptor on osteoclast precursors will trigger osteoclast formation and resorption. The production of reactive oxygen species (ROS) is known to play a crucial role in RANKL-induced osteoclast formation and resorption. G-protein coupled receptor 120 (GPR120) signalling has been shown to affect osteoclast formation, but the exact mechanisms of action require further investigation. RAW264.7 murine macrophages were seeded into culture plates and exposed to the GPR120 agonist, TUG-891, at varying concentrations (20–100 µM) and RANKL to induce osteoclast formation. TUG-891 was shown to inhibit osteoclast formation and resorption without affecting cell viability in RAW264.7 macrophages. TUG-891 further decreased ROS production when compared to RANKL only cells. Antioxidant proteins, Nrf2, HO-1 and NQO1 were shown to be upregulated while the ROS inducing protein, Nox1, was downregulated by TUG-891. Gene silencing revealed that TUG-891 exerted its effects specifically through GPR120. This study reveals that GPR120 signalling may inhibit osteoclast formation and resorption through inhibition on ROS production.

## 1. Introduction

The skeleton is a metabolically active and dynamic tissue which allows for the movement of muscles and protection and support of vital organs [1]. Bone is continuously repaired by a process known as bone remodelling, whereby osteoclasts resorb old bone and osteoblasts form new bone, to maintain the strength and integrity of bone [2]. Osteoclasts are large multinucleated bone cells that are derived from the monocyte-macrophage lineage of haematopoietic stem cells [3]. Osteoclasts are capable of resorbing bone when stimulated by receptor activator of nuclear factor-κB ligand (RANKL) binding to its receptor, RANK, on osteoclast precursors [1]. Over-active osteoclasts can lead to bone degenerative disorders such as osteoporosis. Targeting osteoclast formation represents a viable strategy in the treatment of such bone degenerative disorders.

Studies have shown that reactive oxygen species (ROS) production is crucial for RANKL-mediated osteoclast formation [4,5]. RANK, a member of the tumour necrosis factor (TNF) family, shares many features with other TNF receptors such as the increased ROS production upon receptor binding [6]. When RANKL binds to RANK, the adapter molecule TNF receptor associated factor 6 (TRAF6) is recruited, which results in the activation of nuclear factor κB (NF-κB) and mitogen activated protein kinases (MAPKs) signalling pathways, ultimately leading to the activation of the master regulator of osteoclast formation, nuclear factor of activated T-cells 1 (NFATc1) [7]. ROS are believed to be involved in TRAF6 activation of NF-κB and MAPKs [6]. The source of ROS in osteoclasts is not fully understood but nicotinamide adenine dinucleotide phosphate (NADPH) oxidase (Nox) isoforms are thought to be involved [6]. Nox is considered the most important mammalian enzyme in the production of intracellular ROS [8]. Lee et al. have shown that inhibition of Nox1 decreased RANKL-induced osteoclast formation in murine bone marrow macrophages [4]. The transcriptional factor, nuclear factor E2-related factor 2 (Nrf2), which controls the expression of antioxidant proteins such as heme oxygenase-1 (HO-1) and NADPH:quinone reductase (NQO1), has been shown to be downregulated in osteoclast formation [9,10]. RANKL stimulation upregulates kelch-like ECH-associated protein 1 (Keap1), which prevents Nrf2 nuclear translocation and its associated ROS inhibiting effects [10]. By upregulating Nox1 and inhibiting Nrf2 nuclear translocation, RANKL stimulation allows the osteoclast precursor to maintain ROS levels which are necessary for differentiation into bone resorbing osteoclasts.

G-protein coupled receptor 120 (GPR120), also known as a free fatty acid receptor 4 (FFA4), forms part of the rhodopsin-like family of GPRs and serves as a receptor for long-chain polyunsaturated fatty acids (LCUFAs) [11]. Docosahexaenoic acid (DHA), an ω-3 LCPUFA, has been shown to inhibit osteoclast formation and bone resorption in vivo through GPR120 [12]. GPR120 agonists have also been shown to inhibit osteoclast formation through inhibition of NF-κB and MAPK signalling pathways [13]. Indeed, we have previously shown that ω-3 LCPUFAs can exert their anti-osteoclast effects through activation of the GPR120/β-arrestin signalling axis [14]. These recent studies show the potential bone protective effects of GPR120 however, the exact mechanisms of action require further investigation. To our knowledge, the effect of GPR120 signalling on ROS production in osteoclasts has not been studied. In this present study, we aim to further elucidate the mechanisms of action of GPR120 signalling in osteoclasts by examining its effect on ROS production in RANKL-induced osteoclast formation.

## 2. Results

### 2.1. TUG-891 Has No Effect on Cell Viability

**Figure 1:**
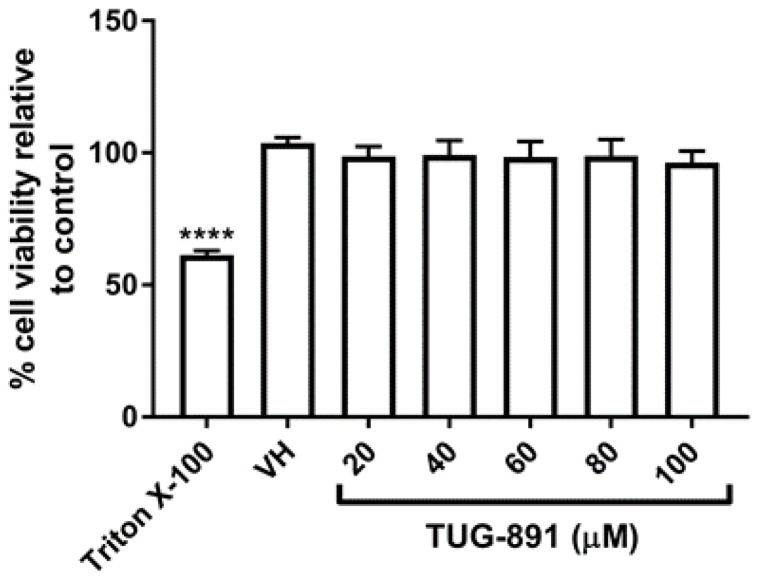
Effect of TUG-891 on cell viability in RAW264.7 macrophages. RAW264.7 macrophages were seeded into 96-well plates and exposed to TUG-891 (20–100 µM) for 48 h. Triton X-100 (0.1%) was used as a positive control for cytotoxicity. Cell viability was determined by a resazurin assay. VH: vehicle control. **** *p* < 0.0001 vs. VH.

A resazurin assay was conducted to test the effects of the highly selective GPR120 agonist, TUG-891, on cell viability in undifferentiated RAW264.7 murine macrophages. Triton X-100 was used as a positive control and was shown to significantly reduce cell viability (Figure 1). TUG-891 showed no effect on cell viability at all the concentrations tested (20–100 µM).

### 2.2. GPR120 Signalling Inhbits Osteoclast Formation and Bone Resorption

**Figure 2:**
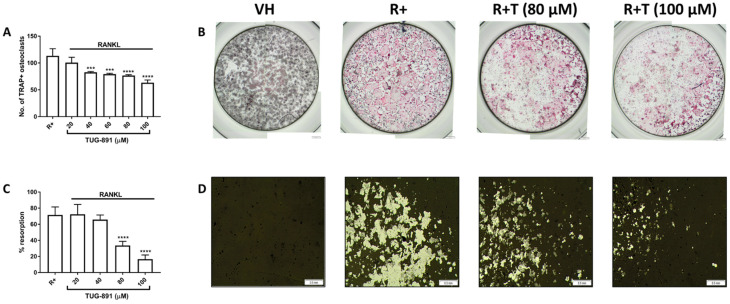
Effect of TUG-891 on osteoclast formation and resorption. (**A**) RAW264.7 macrophages were seeded into 96-well plates and exposed to TUG-891 (20–100 µM) and RANKL (15 ng mL^−1^) together, for 5 days. Medium and all factors were replaced on day 3. Tartrate-resistant acid phosphatase (TRAP) positive cells with 3 or more nuclei were counted. (**B**) TRAP-stained cells were visualized under a light microscope. Cells that stain positive for TRAP appear pink. Scale bar = 500 µm. (**C**) RAW264.7 macrophages were seeded into an osteo assay surface multiwell plate and exposed to TUG-891 (20–100 µM) and RANKL (30 ng mL^−1^) for 5 days. Medium and all factors were replaced on day 3. The resorbed areas were quantified using ImageJ software. (**D**) The plates were stained using a modified von Kossa stain. Resorption pits appear white. Scale bar = 0.5 mm. VH: vehicle control. R+: RANKL only. R+T: RANKL + TUG-891. *** *p* < 0.001, **** *p* < 0.0001 vs. R+.

A tartrate-resistant acid phosphatase (TRAP) cell count was undertaken to determine the effects of TUG-891 on osteoclast formation. TRAP is an enzyme highly expressed in mature osteoclasts [3]. TRAP positive cells with 3 or more nuclei were counted as mature osteoclasts. TUG-891 significantly reduced osteoclast formation (40–100 µM) (Figure 2A). Many large, stained osteoclasts can be seen in RANKL only wells, while fewer cells are seen in the TUG-891 treated wells (Figure 2B).

The effect of GPR120 activation on bone resorption was also evaluated by seeding the cells into an osteo assay surface multiwell plate. After staining the wells using a modified von-Kossa stain, the resorption area was quantified using ImageJ software. TUG-891 (80–100 µM) significantly reduced resorption on the osteo assay surface plate (Figure 2C). Photomicrographs of the wells revealed large, resorption pits in the RANKL only wells (Figure 2D). Resorption pits were greatly reduced in the TUG-891 treated cells.

### 2.3. GPR120 Signalling Promotes Nrf2 Nuclear Translocation, and HO-1 and NQO1 Expression While Inhibiting Nox1 Expression

**Figure 3:**
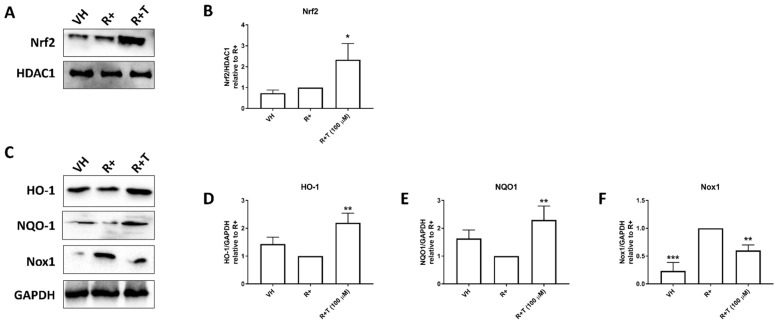
Effect of TUG-891 on expression of proteins involved in ROS production. RAW264.7 macrophages were seeded into 6-well plates and exposed to TUG-891 (100 µM) and RANKL (15 ng mL^−1^). After 24 h a Western blot was performed to determine protein expression. (**A**) Representative Nrf2 and HDAC1 blot. (**B**) Quantification of Nrf2 blots from at least two repeated experiments. (**C**) Representative HO-1, NQO1, Nox 1 and GAPDH blot. (**D**–**F**) Quantification of HO-1, NQO1 and Nox1 blots respectively from at least two repeated experiments. VH: vehicle control. R+: RANKL only. R+T: RANKL + TUG-891. * *p* < 0.05, ** *p* < 0.01, *** *p* < 0.001 vs. R+.

The expression of proteins involved in ROS production was evaluated by Western blot. Cells were treated with RANKL (15 ng mL^−1^) alone or in combination with TUG-891 (100 µM) for 24 h. TUG-891 was shown to significantly increase the nuclear translocation of Nrf2 (Figure 3A,B). HO-1 and NQO1 expression were similarly shown to be increased by TUG-891 (Figure 3C–E). However, TUG-891 was shown to inhibit Nox1 expression. This inhibition was shown to be statistically significant (Figure 3F).

### 2.4. TUG-891 Inhibits Reactive Oxygen Species (ROS) Production through GPR120 Signalling

**Figure 4:**
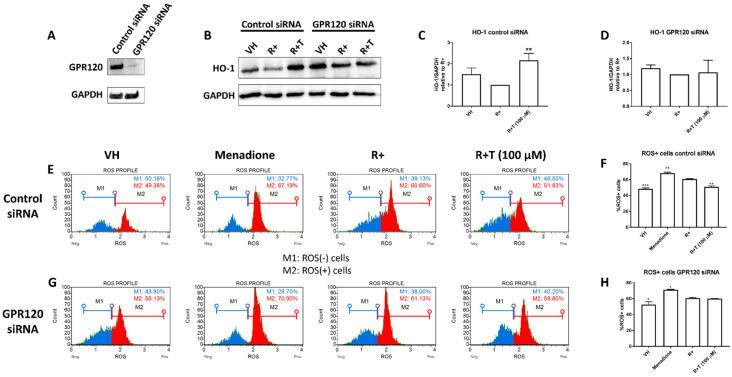
Effect of TUG-891 on HO-1 expression and reactive oxygen species (ROS) production in GPR120 negative cells. RAW264.7 macrophages were seeded into 6-well plates and exposed to TUG-891 (100 µM) and RANKL (15 ng mL^−1^). Cells were exposed to control siRNA or GPR120 siRNA for 24 h. (**A**) GPR120 expression was determined by Western blot. (**B**) The expression of HO-1 was determined. Representative HO-1 and GAPDH blot is shown. (**C**,**D**) Quantification of HO-1 blots for control siRNA and GPR120 siRNA transfected cells respectively from at least two repeated experiments. (**E**) After 48 h intracellular ROS was determined using a Muse^®^ Oxidative Stress Kit Menadione (100 µM) was used as a control for intracellular ROS production. Representative ROS profiles for control siRNA cells are shown. (**F**) Percentage of ROS positive cells in control siRNA cells from two experiments. (**G**) Representative ROS profiles for GPR120 siRNA transfected cells. (**H**) Percentage of ROS positive cells in GPR120 siRNA cells from two experiments. VH: vehicle control. R+: RANKL only. R+T: RANKL + TUG-891. * *p* < 0.05, ** *p* < 0.01, *** *p* < 0.001 vs. R+.

To show that the effects of TUG-891 were mediated through GPR120, gene silencing was conducted. GPR120 siRNA reduced GPR120 expression compared to the control siRNA (Figure 4A). TUG-891 was shown to increase HO-1 expression in control siRNA cells (Figure 4B,C). However, GPR120 silencing prevented the stimulatory effects of TUG-891 on HO-1 (Figure 4D).

To determine the effects of GPR120 activation on ROS production, an oxidative stress assay was conducted. The Muse^®^ Oxidative Stress Kit and a Muse^®^ Cell Analyzer were used to quantify intracellular ROS production. Menadione (100 µM) was used as a control for ROS induction and increased the percentage of ROS positive cells. In control siRNA cells, the percentage of ROS positive cells was significantly decreased by TUG-891 compared to the RANKL only control (Figure 4E,F). However, when GPR120 was silenced, the percentage of ROS positive cells was unchanged by TUG-891 when compared to RANKL only cells (Figure 4G,H).

## 3. Discussion

GPR120 is an ω-3 LCPUFA receptor that has been shown to mediate the anti-inflammatory effects of ω-3 LCPUFAs [15]. Numerous studies have reported on the bone protective effects of ω-3 LCPUFAs [16,17,18,19]. This has led to an interest in understanding the role of GPR120 in mediating the effects of ω-3 LCPUFAs in bone. Kishikawa et al. have reported that the ω-3 LCPUFA, DHA, inhibited inflammation induced osteoclast formation and resorption through GPR120, in vivo [12]. We have previously reported that ω-3 LCPUFAs utilize the GPR120/β-arrestin 2 signalling axis to mediate their inhibitory effects on osteoclasts and to stimulate osteoblast gene expression [14]. Similarly, in this present study we report that the GPR120 agonist, TUG-891, inhibited osteoclast formation and bone resorption in RAW264.7 murine macrophages. TUG-891 has previously been shown to stimulate osteoblast differentiation and protect against ovariectomy induced bone loss [20]. Kim et al. have reported that the GPR120 agonist, GW9508, can inhibit osteoclast formation and resorption through inhibition of NF-κB and MAPK signalling pathways [13]. However, the effect of GPR120 signalling on ROS production in osteoclasts is unknown.

ROS are known to play an important role in RANKL-induced osteoclast formation [4,5]. ROS may be crucial for TRAF6 mediated activation of NF-κB and MAPK signalling pathways in osteoclasts [6]. Treatment of osteoclast precursors with antioxidants, N-acetyl-L-cystein and glutathione, has been shown to inhibit osteoclast formation through inhibition of NF-κB and MAPK signalling [21]. These studies highlight the crucial role ROS play in the formation and function of osteoclasts. Interestingly, ω-3 LCPUFAs have been shown to exhibit antioxidant properties [22]. Nakamura et al. have shown that the inhibitory effects of the ω-3 LCPUFA, eicosapentaenoic acid (EPA), on Nox expression in smooth muscle cells was cancelled by GPR120 silencing [23]. Similar results have been shown by directly targeting the GPR120 receptor using agonists. TUG-891 was shown to inhibit phorbol-12-myristate 13-acetate (PMA)-induced ROS generation in RAW264.7 murine macrophages [24]. PMA is known to induce ROS generation through Nox [25]. Here, we report that the GPR120 agonist, TUG-891, inhibited ROS generation in RAW264.7 macrophages after RANKL stimulation. We further showed that the inhibitory effects of TUG-891 on ROS production were abrogated in the absence of GPR120. Inhibition of ROS could explain the previously reported inhibitory effects of GPR120 agonists on RANKL-induced NF-κB and MAPK signalling in osteoclasts.

Nox isoforms are the most important ROS producing enzymes [8]. Lee et al. reported that Nox1 and Nox2 are expressed in undifferentiated bone marrow macrophages, while Nox3 and Nox 4 are absent [4]. Studies have also shown that, when pre-osteoclasts are induced with RANKL, Nox2 expression decreases while Nox1 expression increases [26]. Furthermore, Nox1 knockdown has been shown to inhibit RANKL-induced osteoclast formation and MAPK signalling [4]. These results suggest that Nox1 is the key ROS inducing enzyme in osteoclasts. Conversely, Sasaki et al. have reported that bone marrow macrophages from Nox1 knockout mice did not result in decreased ROS production or osteoclast formation compared to wildtype [26]. This may suggest that other Nox isoforms, such as Nox2, can compensate ROS production in the absence of Nox1. Nevertheless, in this study we report that TUG-891 inhibited RANKL-induced Nox1 expression. This suggests that the GPR120 agonist, TUG-891, may abrogate ROS production in osteoclasts through inhibition of Nox1.

Nrf2 is another important transcriptional factor that regulates intracellular ROS in osteoclasts [10]. Sun et al. reported that Nrf2 knockout mice show increases in osteoclasts and bone resorption [27]. Oxidative stress triggers the translocation of Nrf2 into the nucleus, where it will promote the expression of antioxidant genes. RANKL keeps Nrf2 in the cytoplasm by increasing the expression of Keap1, which binds Nrf2 and prevents its translocation into the nucleus [10]. Liu et al. have reported that the GPR120 agonist GW9508 can increase nuclear translocation of Nrf2 in human endothelial cells [28]. Similarly, we have shown in this present study that TUG-891 increased nuclear translocation of Nrf2 in RANKL-induced murine macrophages. Nrf2 regulates the expression of cytoprotective proteins such as HO-1 and NQO1 [29]. Induction of HO-1 has been shown to decrease osteoclast formation and resorption, in vitro and in vivo [30], while activation of NQO1 inhibits RANKL-induced osteoclast formation, in vitro [31]. In this present study we show that TUG-891 increased the expression of HO-1 and NQO-1. This could suggest that GPR120 signalling can further inhibit ROS production in osteoclasts through increased Nrf2 nuclear translocation resulting in increased expression of HO-1 and NQO1. The increase in HO-1 expression induced by TUG-891 was abrogated by GPR120 silencing. This further establishes that the effects of TUG-891 reported in this study were directly through GPR120 signalling.

The results of this study suggest that the GPR120 agonist, TUG-891, may inhibit osteoclast formation and resorption through inhibition of Nox1 expression and stimulation of Nrf2 nuclear translocation, ultimately leading to a decrease in ROS production. GPR120 shows further promise as a drug target in the treatment of bone degenerative diseases.

## 4. Materials and Methods

### 4.1. Materials

Dulbecco’s modified Eagles’ medium (DMEM) was purchased from GIBCO (Invitrogen Corp, Waltham, MA, USA. Foetal bovine serum (FBS) was sourced from Capricorn Scientific (Ebsdorfgrund, Germany). The GPR120 agonist, TUG-891, and all other chemicals of research grade were acquired from Sigma-Aldrich Inc. (St Louis, MO, USA). RANKL was obtained from Research and Diagnostic Systems (R&D Systems, Minneapolis, MN, USA). All cell culture plasticware was supplied by LASEC (Cape Town, South Africa). Antibodies against GPR120, HO-1, NQO1, Nox1, Nrf2, GAPDH and HDAC1 were provided by Abcam (Cambridge, UK).

### 4.2. Cell Cultures

RAW264.7 murine macrophages were acquired from the American Type Culture Collection (ATCC, Rockville, MD, USA) and cultured in DMEM supplemented with 10% foetal bovine serum (FBS) (complete DMEM). The cells were incubated at 37 °C with 5% CO_2_. Cells were passaged by scrapping.

### 4.3. Preparation of TUG-891

TUG-891 was prepared in dimethyl sulfoxide (DMSO) at a stock concentration of 100 mM and stored at −80 °C in the dark until required. Fresh dilutions of the stock solutions were made at the required concentrations (20–100 µM) in complete medium. All experiments contained a vehicle control that consisted of DMSO (0.1%).

### 4.4. Resazurin Assay

A resazurin assay was conducted as previously described [32]. In brief, RAW264.7 murine macrophages were seeded into a sterile 96-well plate in complete DMEM at a density of 5 × 10^3^ cells per well and incubated for 24 h to allow the cells to attach to the plate. Medium was replaced and TUG-891 (20–100 µM) was added to the wells. After 48 h, Triton X-100 (0.1%) was added as a positive control for cytotoxicity. Thereafter, 10% resazurin solution was added to each well. The plates were then left to incubate at 37 °C for 4 h and absorbance was measured using an Epoch Micro-plate spectrophotometer (BioTek Instruments Inc., Winooksi, VT, USA) at a wavelength of 570 nm and using a wavelength of 600 nm as a reference.

### 4.5. Tartrate-Resistant Acid Phosphatase (TRAP) Staining Assay

RAW264.7 macrophages were seeded into a 96-well plate in complete DMEM at a density of 5 × 10^3^ cells per well. TUG-891 (20–100 µM) was added to the wells along with RANKL (15 ng mL^−1^) for 5 days. Medium and all factors were replaced on day 3. At the end of the culture period, the cells were fixed with a 3.7% formaldehyde in phosphate buffered saline (PBS) (*v*/*v*). Thereafter, the cells were stained using napthol/dimethylformamide and sodium nitrite as previously described [33]. Osteoclasts, identified as TRAP positive cells that contain more than three nuclei, were counted and photographed using an Olympus SC30 camera attached to an Olympus BH2 microscope (Olympus, Tokyo, Japan).

### 4.6. Bone Resorption Assay

RAW264.7 murine macrophages were seeded in complete DMEM into a 24-well Corning^®^ Osteo Assay Surface Multiwell Plate (Sigma-Aldrich Inc.) at a density of 1.5 × 10^4^ cells per well. TUG-891 (20–100 µM) and RANKL (30 ng mL^−1^) were added to the wells and the plates were incubated for 5 days. Medium and all factors were replaced on day 3. After the incubation period, all wells were treated with 5% sodium hypochlorite for 5 min to remove the cells. The plate was washed with water, left to dry and then stained using a modified von Kossa stain [34] to visualise the resorption of the bone layer at the bottom of the well. The wells were photographed with an Olympus SC30 camera attached to an Olympus BH2 microscope (Olympus). The percentage resorption was analysed using ImageJ software.

### 4.7. Gene Silencing

RAW264.7 murine macrophages were seeded into a 6-well plate in complete DMEM at a density of 1 × 10^6^ cells per well and allowed to attach overnight. Thereafter, the cells were transfected with MISSION^®^ predesigned control siRNA or GPR120 siRNA using MISSION^®^ siRNA transfection reagent (Sigma-Aldrich Inc.) according to the manufacturers’ instructions. After 24 h, the medium was replaced and the cells were used for Western blotting and oxidative stress experiments.

### 4.8. Nuclear Fractionation

Nuclear protein was isolated as previously described [32]. In brief, RAW264.7 murine macrophages were seeded into a 6-well plate in complete DMEM at a density of 1 × 10^6^ cells per well and allowed to attach overnight. The following day the medium was replaced with complete DMEM that contained RANKL (15 ng mL^−1^) alone or in combination with TUG-891 (100 μM). After 48 h, the cells were lysed using cytoplasmic extraction buffer. After centrifugation, the pellet was resuspended in nuclear extraction buffer and centrifuged again. The supernatant was collected into a fresh tube.

### 4.9. Western Blot

Cells were lysed in ice-cold RIPA buffer supplemented with Protease Inhibitor Cocktail (Sigma-Aldric Inc.) and phenylmethylsulfonyl fluoride (PMSF). Proteins were quantified using a bicinchoninic acid (BCA) protein assay kit (Thermo Scientific, Rockford, IL, USA). Equal amounts of proteins were separated on a 12% polyacrylamide gel and then electro-transferred onto a nitrocellulose membrane using a tris-glycine transfer buffer (25 mM Tris, 20% methanol and 192 mM glycine). Thereafter, the membranes were blocked using a tris-buffered saline (TBS-T) solution containing 5% bovine serum albumin (BSA) for 60 min. The membranes were probed with rabbit polyclonal antibodies against HO-1, NQO1, Nox1, Nrf2, GAPDH and HDAC1 (1:1000) overnight at a temperature of 4 °C. The following day, the membranes were incubated with goat anti-rabbit IgG antibody, HRP-conjugate (1:20,000) (Biorad) secondary antibody for 60 min and then developed using Clarity Western ECL Substrate (Biorad). A ChemiDoc MP (Biorad) was used to visualize the blots and the images were analysed using ImageJ software to determine densitometry data.

### 4.10. Oxidative Stress Assay

After transfection with control or GPR120 siRNA, the medium was replaced with RANKL (15 ng mL^−1^) alone or in combination and TUG-891 (100 μM). Menadione (100 µM) was used as a control to induce intracellular ROS production. After a 48-h incubation, intracellular oxidative stress was analysed using a Muse^®^ Oxidative Stress Kit (Luminex, TX, USA) and a Muse^®^ Cell Analyzer (Luminex) according to the manufacturer’s instructions.

### 4.11. Statistical Analysis

For each assessment, three separate experiments were conducted in triplicate, unless otherwise stated. The data were expressed as a mean ± the standard error of the mean. The data were analysed using GraphPad Prism software (San Diego, CA, USA). A one-way analysis of variance (ANOVA) followed by a Dunnett’s post hoc test was employed to analyse all the data obtained from the experiments performed in this study. All *p*-values < 0.05 were considered significant.

## Data Availability

All data from this study shall be made available upon reasonable request.
